# Chalcogenide Metasurfaces Enabling Ultra‐Wideband Detectors From Visible to Mid‐infrared

**DOI:** 10.1002/advs.202413858

**Published:** 2025-02-19

**Authors:** Shutao Zhang, Shu An, Mingjin Dai, Qing Yang Steve Wu, Nur Qalishah Adanan, Jun Zhang, Yan Liu, Henry Yit Loong Lee, Nancy Lai Mun Wong, Ady Suwardi, Jun Ding, Robert Edward Simpson, Qi Jie Wang, Joel K. W. Yang, Zhaogang Dong

**Affiliations:** ^1^ Institute of Materials Research and Engineering (IMRE) Agency for Science Technology and Research (A*STAR) 2 Fusionopolis Way, Innovis #08‐03 Singapore 138634 Republic of Singapore; ^2^ Singapore University of Technology and Design (SUTD) 8 Somapah Road Singapore 487372 Republic of Singapore; ^3^ Department of Materials Science and Engineering National University of Singapore 9 Engineering Drive 1 Singapore 117575 Republic of Singapore; ^4^ School of Electrical and Electronic Engineering Nanyang Technological University Singapore 639798 Republic of Singapore; ^5^ Department of Electronic Engineering The Chinese University of Hong Kong Sha Tin, New Territories Hong Kong SAR 999077 China; ^6^ University of Birmingham Edgbaston B15 2TT UK

**Keywords:** photodetector, Sb_2_Te_3_ metasurface, thermoelectric effect, ultrawide band

## Abstract

Thermoelectric materials can be designed to support optical resonances across multiple spectral ranges to enable ultra‐wideband photodetection. For instance, antimony telluride (Sb_2_Te_3_) chalcogenide exhibits interband plasmonic resonances in the visible range and Mie resonances in the mid‐infrared (mid‐IR) range, while simultaneously possessing large thermoelectric Seebeck coefficients of 178 µV K^−1^. However, chalcogenide metasurfaces for achieving miniaturized and wavelength‐sensitive ultra‐wideband detectors have not been explored so far, especially with a single material platform. In this paper, Sb_2_Te_3_ metasurface devices are designed and fabricated to achieve ≈97% resonant absorption for enabling photodetectors operating across an ultra‐wideband spectrum, from visible to mid‐IR. Furthermore, relying on linear polarization‐sensitive Sb_2_Te_3_ metasurface, the thermoelectric photodetectors with linear polarization‐selectivity are demonstrated. This work provides a potential platform toward the portable ultrawide band spectrometers without requiring cryogenic cooling, for environmental sensing applications.

## Graphical abstract

Thermoelectric Sb_2_Te_3_ metasurfaces are engineered to support resonant absorption across a broad spectral range, enabling photodetectors with ultra‐wideband and polarization‐sensitive capabilities. This single‐material platform offers a promising approach for developing portable spectrometers, eliminating the need for cryogenic cooling and paving the way for advanced environmental sensing applications.

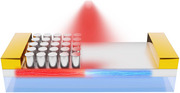

## 1 Introduction

Metasurfaces, consisting of sub‐wavelength nanostructures arranged in a 2D manner, offer capabilities for manipulating light‐matter interactions. It therefore enables a powerful platform to achieve compact optical devices for various applications, such as ultrathin optical lens,^[^ ^1^ ^]^ nanostructured color pixels,^[^ ^2^ ^]^ communication,^[^ ^3^ ^]^ fluorescence enhancements,^[^ ^4^ ^]^ optical nonlinearity,^[^ ^5^ ^]^ anti‐counterfeiting,^[^ ^4^, ^6^ ^]^ energy harvesting,^[^ ^7^ ^]^ and miniaturized optical detectors.^[^ ^8^ ^]^ For example, due to its resonant interaction with light, miniaturized optical detectors that are integrated with semiconductor metasurfaces are able to detect the multidimensional characteristics of light, such as wavelength,^[^ ^8d^ ^]^ polarization,^[^ ^9^ ^]^ and angle.^[^ ^10^ ^]^ Nevertheless, these detection mechanisms are usually constrained by their bandgap to a specific working spectral range, either to visible, near‐infrared (NIR), or mid‐infrared (mid‐IR) range.

On the other hand, photodetection based on the thermoelectric effect has the intrinsic advantage of a wide working wavelength range. For example, aluminum plasmonic metasurfaces fabricated on top of a commercial photo‐thermoelectric (PTE) detector enhance the light absorption and photodetector sensitivity for both visible and near‐IR spectra.^[^ ^11^ ^]^ Other thermoelectric material platforms,^[^ ^12^ ^]^ such as 2D MoS_2_, WSe_2,_ and PdSe_2_, have been explored,^[^ ^13^ ^]^ for the integration with either nanoantenna^[^ ^14^ ^]^ or waveguide,^[^ ^15^ ^]^ with reports of relatively low light absorptance.^[^ ^16^ ^]^ For instance, the absorptance of graphene can be as low as 2.3%.^[^ ^17^ ^]^ An alternative approach uses the lateral *p*‐*n* heterojunction based on Bi_2_Te_2_Se‐Sb_2_Te_3_, with both the flat film design^[^ ^13a^ ^]^ and grating structures with guided‐mode resonances,^[^ ^18^ ^]^ mostly working in the visible spectrum. Some of us have recently demonstrated PTE detectors integrated with an optical cavity for mid‐IR photodetection.^[^ ^19^ ^]^ However, the full potential of thermoelectric material flatform for ultra‐wideband light detection, especially from visible to mid‐IR, has not been explored.

In this paper, we utilize the optical and thermoelectric properties of a chalcogenide material, antimony telluride (Sb_2_Te_3_) to design ultra‐broadband metasurface detectors operating at room temperature. We leverage its interband plasmonic resonance, showing examples for the visible spectrum and Mie resonance in the mid‐IR spectrum, via exploring their respective resonant absorption capabilities. The photodetection mechanism involves photo‐absorption that leads to local temperature rise and a measurable voltage across the material due to its thermoelectric properties. By leveraging the Seebeck coefficient of ≈178 µV K^−1^ and the dielectric function supporting Sb_2_Te_3_ Mie resonances^[^ ^20^ ^]^ and plasmonic resonances, we designed ultra‐wideband metasurfaces with resonant wavelengths and achieved a maximum absorptance of ≈97% at ≈532 nm. Additionally, the Sb_2_Te_3_ metasurface was employed to create linear polarization‐selective ultrawide band thermoelectric photodetectors. This research contributes to the integration of thermoelectric and optical functionalities through nanofabrication techniques. It provides precise control of device absorptance over wavelength and polarization, aiming to improve photodetection applications.

## 2 Results

**Figure 1:**
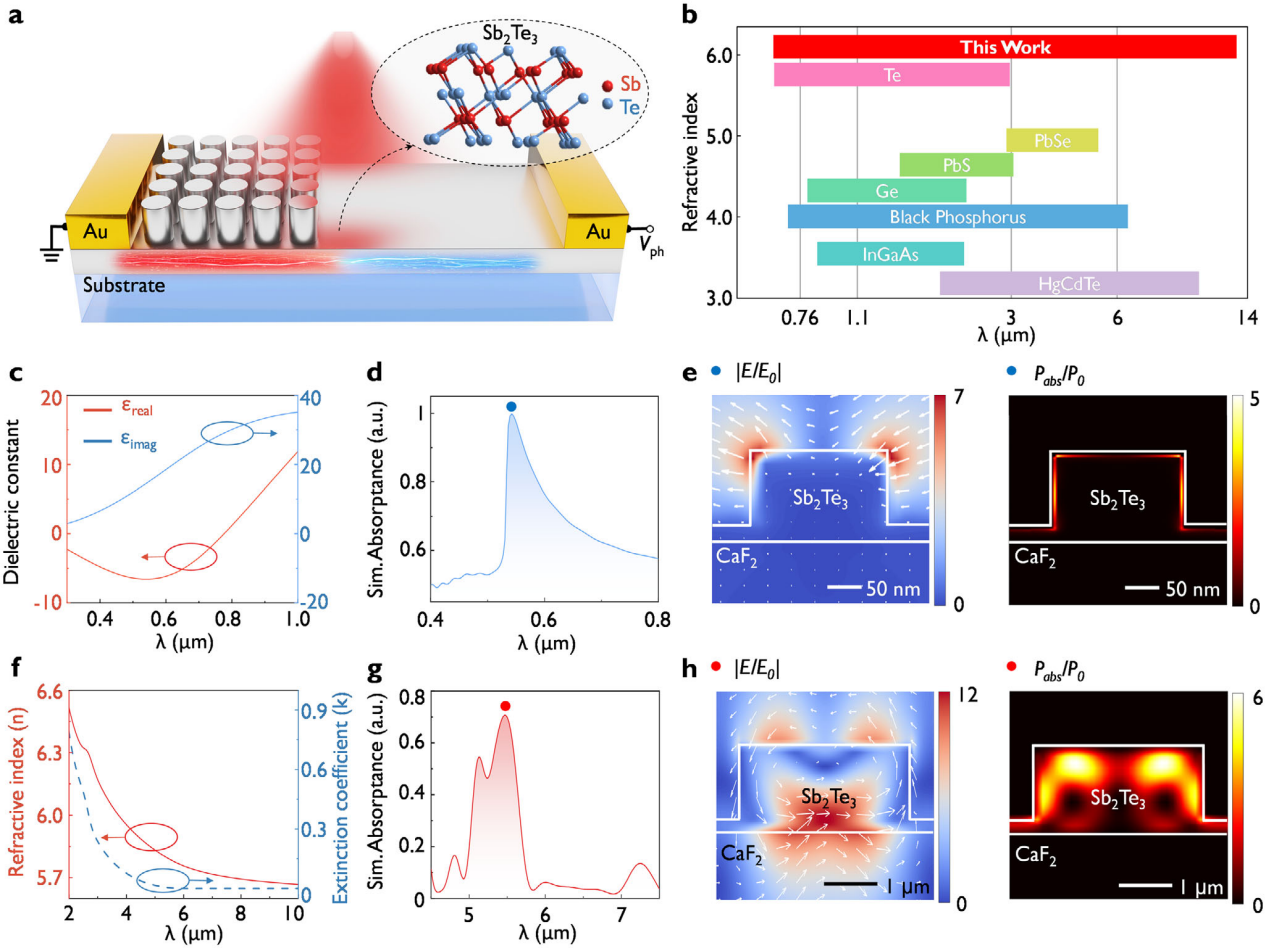
Sb_2_Te_3_ chalcogenide metasurfaces for enabling ultra‐wideband photodetectors due to the resonant absorption from visible to mid‐IR spectrum. a) Schematic of the Sb_2_Te_3_ metasurface photodetectors consisting of nanodisks being etched into the Sb_2_Te_3_ film close to the left electrode. The enhanced absorptance at the metasurface region will lead to a temperature difference upon light absorption and a thermoelectric‐induced measurable voltage readout across the electrodes. The insert shows the crystal structure of rhombohedral‐phase Sb_2_Te_3_. b) A summary and benchmarking on the operational wavelength range of common material platforms for photodetectors. This work (Sb_2_Te_3_) exhibits a high refractive index in the mid‐infrared. c) The dielectric function of Sb_2_Te_3_ film in the visible region, where the real part is negative in this band. d) Visible absorptance spectrum of the Sb_2_Te_3_ nanostructures (diameter: 200 nm, height: 150 nm, and pitch: 532 nm). e,h) Simulated electric field distributions normalized to incident electric field (|*E/E_0_* |) distribution and absorption intensity distribution (|*P_abs_/P_0_* |) across a cross‐section of the visible (blue point) and mid‐IR (redpoint) Sb_2_Te_3_ nanodisk. f) The refractive index (*n*) and extinction coefficient (*k*) of Sb_2_Te_3_ in mid‐IR range. g) Simulated mid‐IR absorptance spectrum of the Sb_2_Te_3_ nanostructures (diameter: 2.0 µm, height: 900 nm and pitch: 3.0 µm).

**Figure** **1**a presents the schematic of our designed thermoelectric photodetector that leverages the combined thermoelectric and optical properties of Sb_2_Te_3_ nanostructures (see Figure S1, Supporting information for detailed schematic). The illustration shows the crystal structure of Sb_2_Te_3_, and the material characterization results are shown in Figure S2 (Supporting information). The detector consists of a Sb_2_Te_3_ strip on CaF_2_ substrate, being connected to Au electrodes at both ends for electrical voltage readout. To generate a temperature gradient upon light illumination, Sb_2_Te_3_ metasurface is patterned only at one end of the strip. This metasurface enhances light absorptance and thus the metasurface will be hotter than the unpatterned Sb_2_Te_3_ side, inducing a potential difference. To achieve wavelength‐selective detection, Sb_2_Te_3_ nanostructures with varying structural parameters can be designed, enabling resonant light absorption to specific wavelengths across a broad spectrum. Figure 1b presents the working wavelength range of our designed Sb_2_Te_3_ metasurface detector, spanning a larger range than bandgap‐limited devices of other photodetector material platforms.^[^ ^21^ ^]^ Noted that the responsivity of thermoelectric photodetectors is notoriously low, but remains constant throughout,^[^ ^22^ ^]^ as summarized in Table S1 (Supporting information). First, we discuss the plasmonic resonance characteristics of Sb_2_Te_3_ in the visible spectrum, where Figure 1c presents the dielectric constant. The corresponding refractive index (*n*) and extinction coefficient (*k*) of Sb_2_Te_3_ in the visible range are depicted in Figure S3 (Supporting information). At wavelengths ranging from 300 to 760 nm, the real part of the dielectric constant is negative, indicating a plasmonic range due to interband transitions.^[^ ^23^ ^]^ For instance, Figure 1d presents the simulated absorption spectrum of Sb_2_Te_3_ nanodisk arrays, which shows a plasmonic resonance with a peak at 532 nm. At this wavelength, the electric field near the material surface is enhanced by ≈7, as illustrated in Figure 1e (left panel). This enhanced field further promotes the interaction between light and Sb_2_Te_3_, thereby increasing the absorption effect, as shown in Figure 1e (right panel). Characteristic of plasmonic‐like resonances, the absorption is constrained to the surface of the nanostructure. Note that the dielectric constant of our grown Sb_2_Te_3_ thin film is different from the one as reported in the literature,^[^ ^23b^ ^]^ primarily due to variations in deposition conditions, such as film thickness, growth temperature, pressure, and gas flow rate. These factors influence the crystallinity, grain size, and defect density of the grown Sb_2_Te_3_ film, leading to observable differences in the refractive index (*n*) and extinction coefficient (*k*).

Next, we discuss the Mie resonance characteristics of Sb_2_Te_3_ at the mid‐IR range due to its high refractive index, which enables the mid‐IR field localization due to the high Q‐factor resonances. For instance, Figure 1f presents the refractive index (*n*) and extinction coefficient (*k*) in the mid‐IR range, where it has a high refractive index of 5.7–6.5 at the wavelength range of 2–10 µm. Finite‐difference time‐domain (FDTD) simulations were carried out for mid‐IR Sb_2_Te_3_ nanodisk arrays (diameter: 2.0 µm, height: 900 nm and pitch: 3.0 µm), where the simulated absorptance spectrum is shown in Figure 1g. The absorptance peak at 5.8 µm indicates resonant absorption, demonstrating its capacity for mid‐IR resonant interactions, where the corresponding electrical field amplitude (|*E/E_0_* |) and absorption distribution are shown in Figure 1h. The high electric and magnetic fields within the Sb_2_Te_3_ disk indicate strong intensity localization, exhibiting characteristics of Mie resonance. In addition, we need the Sb_2_Te_3_ base to conduct electricity, optimized to achieve the best absorption of 150 nm thick (see Figure S4, Supporting information). At the same time, this Sb_2_Te_3_ base layer also introduces the Fabry‐Pérot (FP) resonance due to its high refractive index and specific thickness. To better study the optical response mechanism of the metasurface, multipolar decomposition analysis was conducted, as shown in Figure S5 (Supporting information), where magnetic dipole (*MD*) is the dominant component, followed by electrical dipole (*ED)* contributions at 5.8 µm. Here, we also would like to mention that In the near‐IR region, Sb_2_Te_3_ acts as a lossy dielectric material without effective structural resonances, leading to inferior detector performance compared to its capabilities in the visible and mid‐IR spectra, as indicated by the experimental measurements of the complex refractive index *n* and *k* as shown in Figure S6 (Supporting information). At the same time, due to intrinsic material absorption, Sb_2_Te_3_ should be still able to work as PTE detector at near‐IR wavelength region.

**Figure 2:**
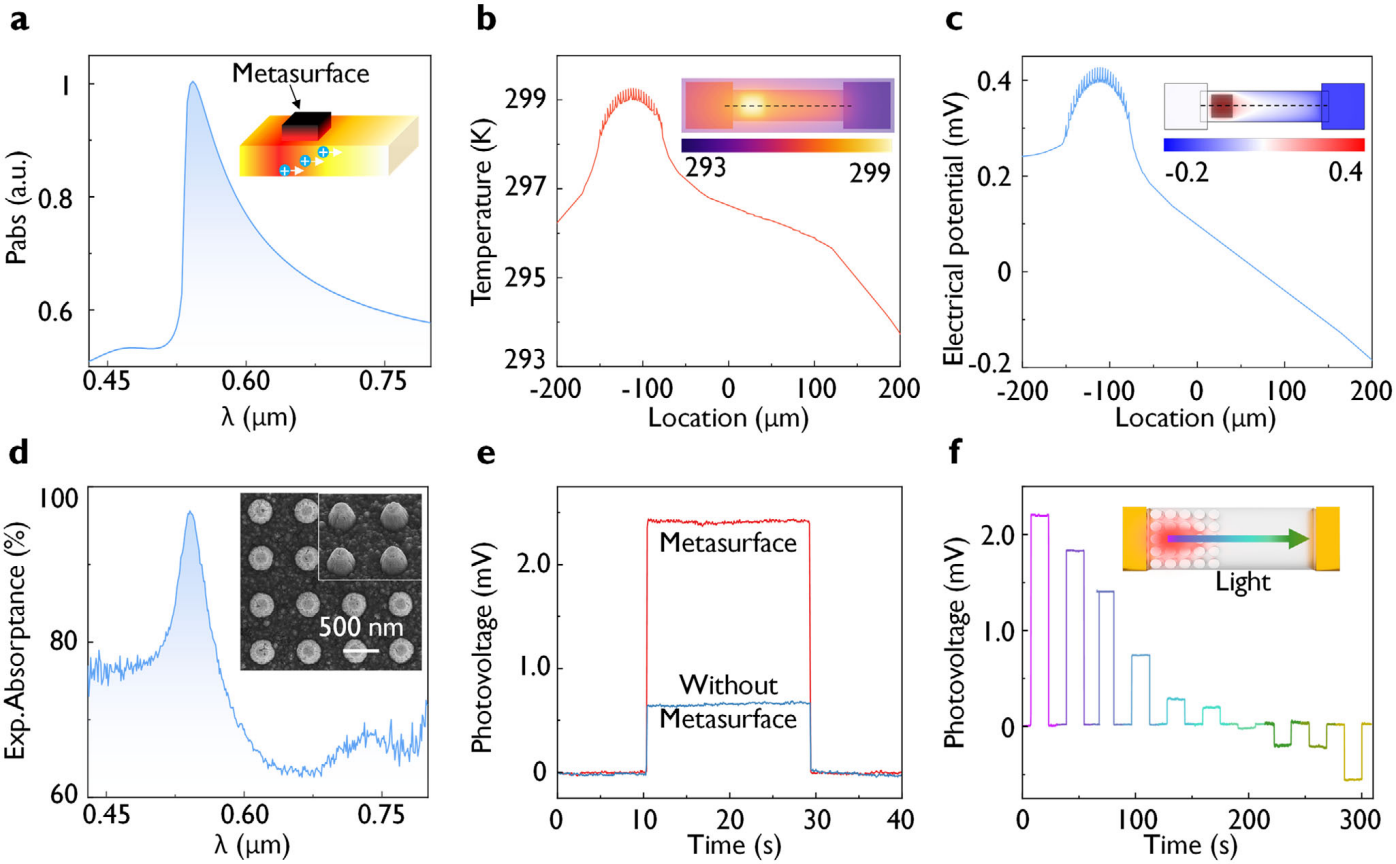
Chalcogenide metasurface photodetectors based on interband plasmonics of Sb_2_Te_3_ at visible wavelength. a) Simulated absorption power density with an incident power of 0.1 mW for the visible light metasurface. Insert shows the schematic illustration of the photo‐thermoelectric (PTE) conversion process in the metasurface. b) Simulated temperature gradient with an input heating power of 0.1 mW and corresponding temperature map for the thermoelectric detector with the Sb_2_Te_3_ metasurface. c) Simulated resulting electric potential and corresponding potential map. Metasurface occupies an area of 75 × 75 µm^2^ and consists of disks of diameter 200 nm, height 150 nm, and pitch 532 nm. d) Measured absorptance spectrum of the designed visible Sb_2_Te_3_ photodetector (disk diameter 200 nm, height 150 nm, pitch 532 nm, base Sb_2_Te_3_ film thickness of 150 nm). The visible Sb_2_Te_3_ nanostructures and the base layer are shown insert. e) Measured photovoltages from the Sb_2_Te_3_ detector when the 532 nm laser illuminates the metasurface and flat substrate regions respectively. The source meter unit is grounded at the electrode near the metasurface. f) Measured photovoltage when the focused 532 nm laser spot is scanned from the metasurface to the flat region. The incident optical power is 0.1 mW.

According to the photothermoelectric (PTE) effect, the photoresponse can be divided into two distinct processes: the photothermal effect and the thermoelectric effect, as shown in the inset of **Figure** **2**a. When active materials like Sb_2_Te_3_ absorb light, a temperature difference is generated between the two ends of the device. Most carriers (holes in *p*‐type material Sb_2_Te_3_) are driven from the hot end to the cold end due to the Seebeck effect, resulting in a potential difference across the channel. For photothermal conversion, we enhance local heating through the hybrid resonances of the metasurface. In the visible wavelength range, Sb_2_Te_3_ nanostructures can be designed to exhibit interband plasmonic resonance, with the simulated absorption power density shown in Figure 2a. The designed Sb_2_Te_3_ metasurface demonstrates the strongest absorption at 532 nm. We further simulated the temperature distribution of thermoelectric detectors with and without the Sb_2_Te_3_ metasurface under an input heating power of 0.1 mW. As shown in Figure 2b, the temperature difference within the detector device becomes more pronounced under the Sb_2_Te_3_ metasurface, reaching a maximum of 6.0 Kelvin due to the enhanced electric field in the metasurface region. In contrast, without the metasurface (Figure S7, Supporting information), the temperature distribution in Sb_2_Te_3_ is relatively uniform, with a temperature difference of 1.0 Kelvin. Figure 2c and Figure S8 (Supporting information) show the simulated potential distribution of these thermoelectric detectors under the same input power. In the absence of the metasurface, the potential distribution is relatively flat, with the highest potential of 0.20 mV at the left end. On the Sb_2_Te_3_ metasurface, as holes migrate to the cold end on the right side, the potential gradient is enhanced, with the lowest potential on the metasurface reaching 0.40 mV. The simulations further reveal that the temperature difference is proportional to the absorbed power, as shown in Figure S9 (Supporting information). We can also note that if a very small gold electrode is precisely placed in the hottest region of the metasurface, while another electrode is placed farther away at room temperature, the responsiveness is expected to improve. The analysis indicates that the Sb_2_Te_3_ metasurface enhances both the temperature gradient and the potential gradient, thereby improving photothermoelectric conversion efficiency and providing an effective approach to optimizing the performance of photodetectors.

Figure 2d presents the experimental absorption results of the visible metasurface design, with an absorption rate of 97% at 532 nm. To demonstrate the enhanced PTE effect on metasurface photodetectors, we conducted photovoltage tests on two types of detectors, with and without metasurfaces. As shown in Figure 2e, when the 532 nm laser is turned on, a voltage step of 2.4 mV is observed in the device with a metasurface; in comparison, when the laser illuminates the device with the flat Sb_2_Te_3_ film, a voltage step of only 0.65 mV is detected. In other words, the corresponding responsivities are 24 and 6.5 V W^−1^ respectively, indicating a 4‐fold increase in responsivity. The external quantum efficiency (EQE) is calculated to be ≈56%. When the laser irradiates from the metasurface region to the flat region, the photovoltage changes from positive to negative, as shown in Figure 2f. Under 532 nm laser illumination at the metasurface, the voltage reaches 2.2 mV. As the focused laser spot moves toward the flat region, the voltage gradually shifts, ultimately reaching 0.5 mV at the flat region. This measurement indicates a 4‐fold voltage enhancement effect at the metasurface due to the resonance absorption, where Sb_2_Te_3_ nanostructures play a crucial role in thermoelectric detection, enhancing the thermoelectric response through interband plasmonic resonance.

**Figure 3:**
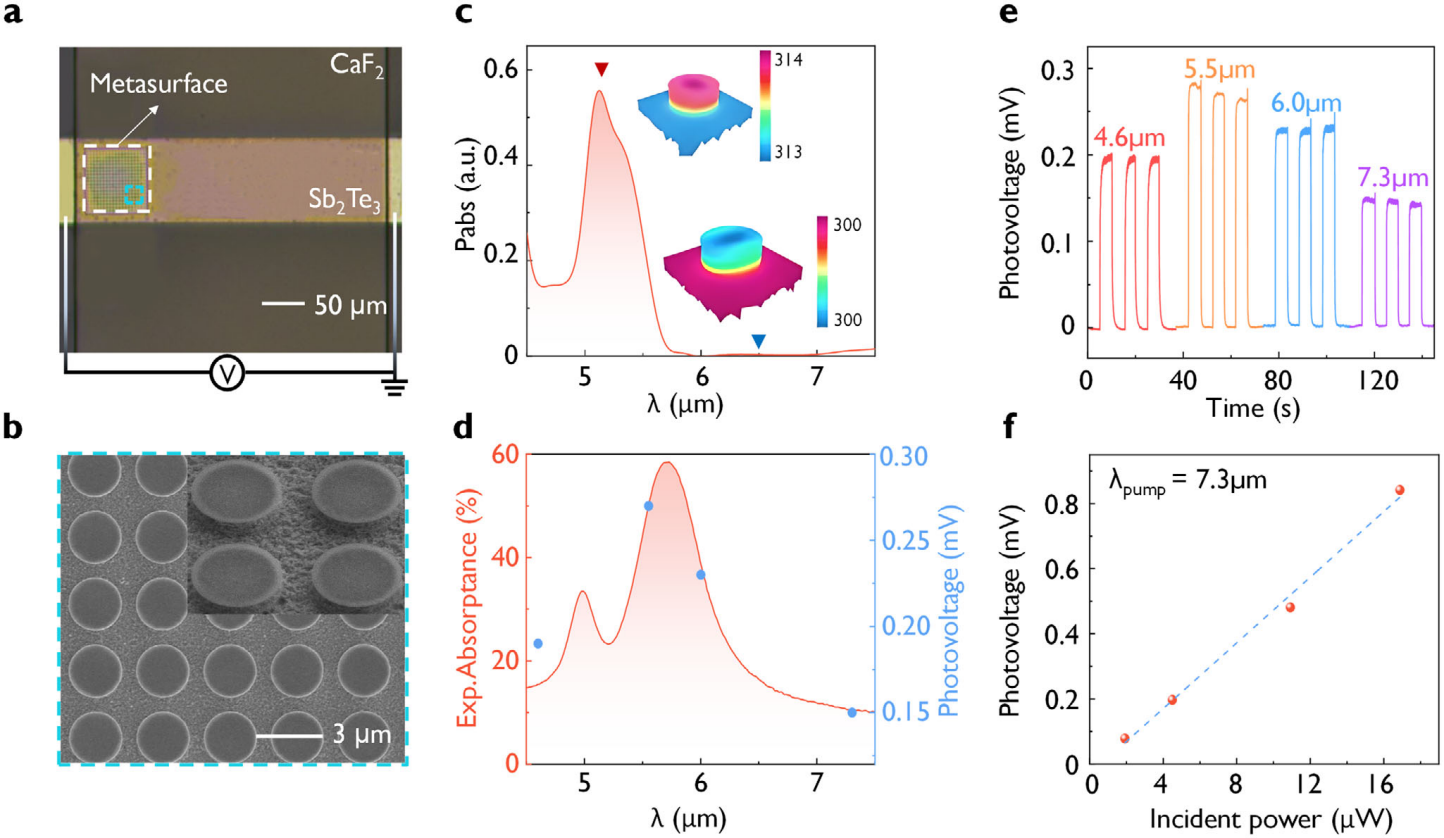
Sb_2_Te_3_ mid‐IR photodetector with hybridized resonance due to Mie and Fabry–Pérot (FP) cavities. a) Optical microscope image of the Sb_2_Te_3_ photodetector. The square region on the left end is the patterned metasurface regions. b) SEM image of the fabricated mid‐IR Sb_2_Te_3_ metasurface. c) Simulated absorption power density with an incident power of 0.1 mW for the mid‐IR metasurface. Insets show the temperature profile of the metasurface under corresponding wavelength conditions. d) Measured absorptance spectrum of the mid‐IR Sb_2_Te_3_ photodetector (disk diameter 2.0 µm, height 0.9 µm, pitch 3.0 µm, base Sb_2_Te_3_ film thickness of 150 nm). e) Measured photovoltages with an incident laser power of 4.5 µW at different wavelengths (4.6, 5.5, 6.0, 7.3 µm). f) Power‐dependent photovoltage under illumination of incident optical powers from 1.9 to 16.8 µW.

Next, we investigate the fabrication of wavelength‐selective thermoelectric detectors based on hybrid Mie‐Fabry‐Pérot (Mie‐FP) resonances using a high refractive index of Sb_2_Te_3_ in the mid‐IR region. The nanofabrication process for Sb_2_Te_3_ thermoelectric detectors is shown in Figure S10 (Supporting information). Figure S11 (Supporting information) shows the fabricated Sb_2_Te_3_ metasurface detector on a CaF_2_ substrate, where it is connected to a printed circuit board (PCB) for measurement. By designing Sb_2_Te_3_ metasurfaces with different geometrical dimensions, multiple thermoelectric detectors can be fabricated on a single PCB board. **Figure** **3**a presents the optical microscope image of showing mid‐IR Sb_2_Te_3_ detector, which consists of a narrow strip of a width of 100 µm and a length of 400 µm. The enhanced absorption at the metasurface region increases the temperature gradient to result in a higher voltage. In addition, for the purpose of hot carrier transportation and the subsequent formation of voltage across the two electrodes, the mid‐IR Sb_2_Te_3_ metasurface was formed by partial etching to a depth of 750 nm, leaving a 150‐nm‐thick base layer. Figure 3b shows the SEM images of the mid‐IR Sb_2_Te_3_ nanostructures with a base layer.

Figure 3c shows the simulated absorption power density of the Sb_2_Te_3_ metasurface in the mid‐IR region, with the strongest absorption observed at 5.3 µm. The insets illustrate the simulated temperature profiles at 5.3 and 6.5 µm, where the absorption power density at 5.3 µm is higher, leading to a temperature gradient of up to 0.35 Kelvin µm^−1^, indicating the effective conversion of incident light into heat by the metasurface. Figure 3d presents the measured absorptance of the sample, reaching 60% at 5.9 µm. By varying the diameter of the Sb_2_Te_3_ structures from 1.4 to 2.0 µm, there exists a dual absorption modulation, ranging from 4.3 to 5.9 µm (see details in Figure S12, Supporting information). To evaluate the wavelength‐selective detection capability and high sensitivity at room temperature, a series of photoelectric tests were performed using a series of quantum cascade lasers (QCL, Daylight Solutions, MIRcat) of different wavelengths and intensities. For instance, Figure 3e shows the response of the Sb_2_Te_3_ metasurface device to the laser wavelength of 4.6, 5.5, 6.0, and 7.3 µm, with a constant power of 4.5 µW. Notably, the voltage response to the 5.5 µm laser is the highest, reaching 300 µV, and the responsivity reached to 67 V W^−1^, due to the peak absorption of the device at this wavelength. The calculated EQE is 30%, with a measured response speed of ≈160 ms (see Figure S13, Supporting information). In other words, our Sb_2_Te_3_ detector can achieve wavelength‐sensitive detection in the mid‐IR range. This demonstrates the potential for integrating multiple wavelength‐selective metasurfaces into a single detector to enable multi‐wavelength detection within a compact design.^[^ ^24^ ^]^ Additionally, the impact of incident power level on the photovoltage was analyzed, as shown in Figure 3f. The photovoltage varies with increasing laser power. Through linear fitting, we obtained *R^2^* = 0.997 and the *V_PTE_* = 0.05*P_Incident_ –* 0.031, indicating a linear relationship between the photovoltage and incident light power. This trend highlights the amplification of the photoelectric effect with increased laser power, leading to increased absorption by the Sb_2_Te_3_ nanostructures, thereby increasing the thermal gradient and consequently the photovoltage.

**Figure 4:**
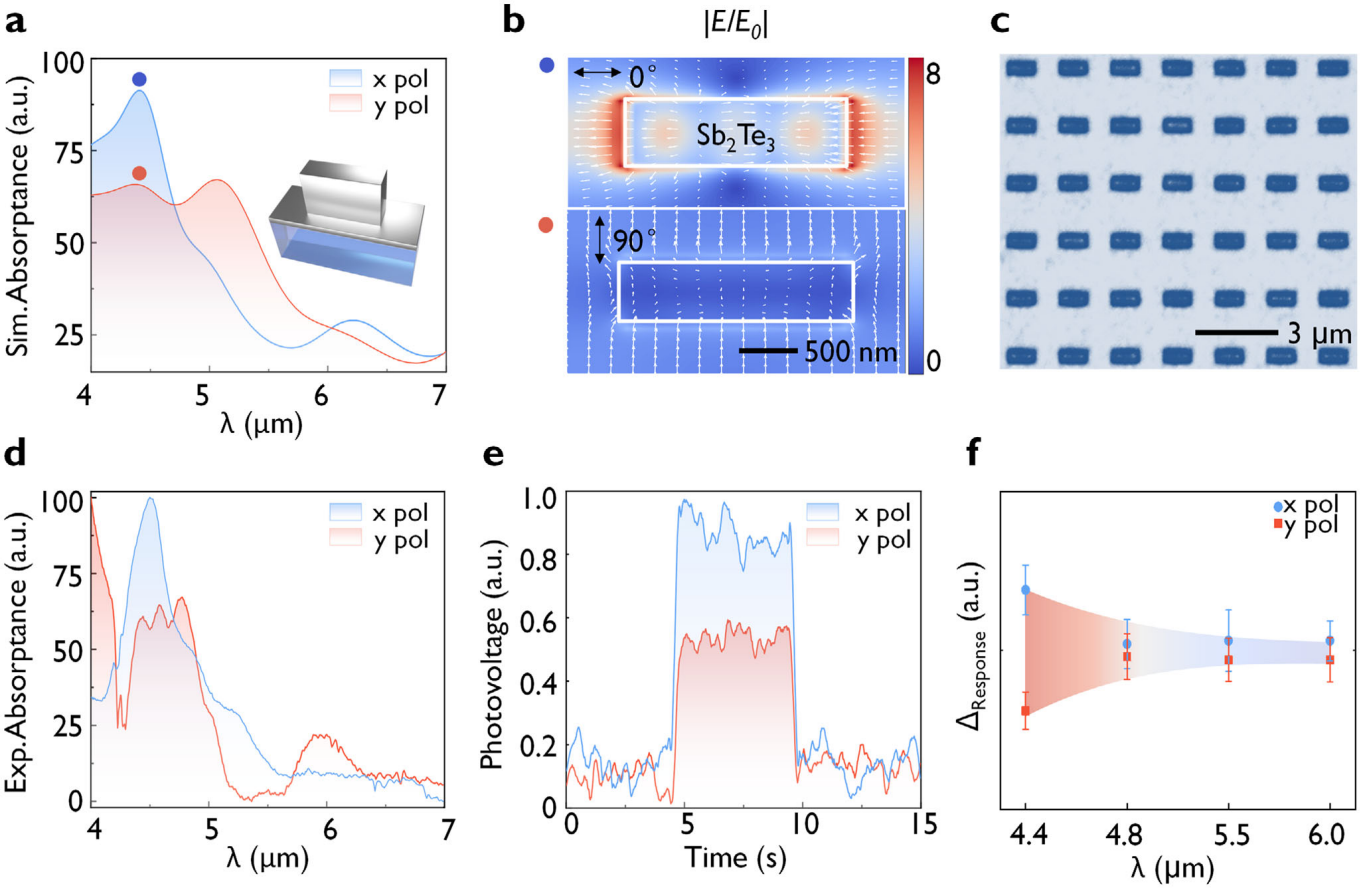
Linear polarization‐sensitive Sb_2_Te_3_ mid‐IR photodetector. a) Simulated polarization‐dependent absorptance. Insert shows the schematic of polarized Sb_2_Te_3_ infrared nanostructure. b) Simulated electric field distributions (|*E/E_0_* |) for *x*‐ and *y*‐polarizations. c) Optical microscope image of the polarization‐dependent Sb_2_Te_3_ metasurface. d) Measured polarization‐dependent absorptance by Fourier Transform Infrared Spectroscopy (FTIR). e) Photovoltage measurements when the laser shines on the nanostructure under 4.5 µm laser with different polarization. f) The polarization standard deviation of the photovoltage measurement when the polarization‐sensitive Sb_2_Te_3_ mid‐IR photodetector is illuminated by laser working at different wavelengths.

To explore whether its high refractive index can enable high‐dimensional optical information detection, and polarization‐sensitive metasurface was investigated. The structure parameters of the Sb_2_Te_3_ rectangular array were optimized using the Particle Swarm Optimization (PSO) algorithm (see details in Figure S14, Supporting information),^[^ ^25^ ^]^ yielding a length of 2.0 µm, width of 1.0 µm, and a period of 3.0 µm. The Sb_2_Te_3_ film base layer was also 150 nm. **Figure** **4**a presents the simulated absorption spectrum, showing an absorption difference at 4.5 µm. And by changing the polarization Angle at this wavelength, we can see significant polarization selectivity (as shown in Figure S15, Supporting information). The inset of Figure 4a illustrates the nanostructure of the linear polarization‐sensitive mid‐infrared photodetector based on Sb_2_Te_3_. Additionally, the simulated electric field distributions (|*E/E_0_* |) and absorption intensity distributions (|*P/P_0_* |) under different polarizations are shown in Figure 4b and Figure S16 (Supporting information), where the electric field amplitude and absorption intensity are higher at 0° polarization compared to 90° polarization. Figure 4c presents the optical image of the fabricated Sb_2_Te_3_ polarization‐dependent metasurface array, clearly demonstrating the well‐formed morphology of the Sb_2_Te_3_ rectangular array. The measured spectra under different polarizations, as shown in Figure 4d, consistent with the design expectations.

The schematic diagram of the experimental device for photovoltage measurement is shown in Figure S17 (Supporting information). The corresponding photovoltage indicates a polarization ratio (*PR*, calculated as *PR = V_max_/V_min_*) of 1.09, demonstrating a response consistent with the observed absorption contrast. To verify the polarization sensitivity of the metasurface to different laser wavelengths, lasers with wavelengths of 4.5, 4.8, 5.5, and 6.0 µm were used to irradiate the sample and measure the corresponding photovoltage. As shown in Figure 4f, the 4.5 µm laser exhibited the highest polarization sensitivity and absorption difference, corresponding to the metasurface absorption measured in the experiment. This measurement demonstrates that by designing different Sb_2_Te_3_ metasurfaces, wavelength, and linear polarization‐selective absorption in the mid‐IR region at room temperature can be achieved.

## 3 Conclusion

We demonstrate that the chalcogenide antimony telluride (Sb_2_Te_3_) is a multifunctional material platform for photodetection applications, due to its excellent thermoelectric properties as well as its rich optical characteristics across multiple spectral ranges, from visible to mid‐IR. Through the systematic designs, we have demonstrated the nanostructured Sb_2_Te_3_ metasurface detectors, working in both visible and mid‐IR ranges, where the polarization‐sensitive characteristic can also be enabled by designing the polarization‐sensitive metasurface. This work advances the integration of thermoelectric and optical functionalities of Sb_2_Te_3_ through nanofabrication, providing a novel platform for single‐material ultra‐broadband metasurfaces and offering new directions for enabling the miniaturized on‐chip detection of the higher‐dimensional characteristics of light across an ultra‐wideband spectrum wavelength range.

## 4 Experimental Section

### Sb_2_Te_3_ Sputtering via Radio Frequency (RF) Sputtering

A Sb_2_Te_3_ film (1µm) was deposited on CaF_2_ substrate (10mm × 10 mm × 0.5 mm) by using radio frequency (RF) sputtering. The film was deposited from a 2‐inch diameter Sb_2_Te_3_ target (purity > 99.9%) with a power of 30 W and a pressure of 3.7 mTorr pure Ar at room temperature. The deposition rate was 4.725 nm min^−1^.

### Nanopatterning of Sb_2_Te_3_ Metasurfaces

A ≈30 nm Hydrogen silsesquioxane (HSQ) mask was created on the surface of Sb_2_Te_3_/CaF_2_ sample by using an electron beam lithography (EBL, Elionix ELS‐7000). First, HSQ resist (Dow Corning XR‐1541‐006) was spin‐coated onto a cleaned sample at 3000 round‐per‐minute (rpm) to obtain a HSQ thickness of ≈100 nm. Then, electron beam exposure was carried out with an electron acceleration voltage of 100 keV and a beam current of 500 pA. After completing the exposure, the pattern was immediately developed by NaOH/NaCl salty solution (1% wt./4% wt. in de‐ionized water) for one minute. After that, the sample was flushed by de‐ionized water for one minute to stop the development. Finally, the sample was rinsed by using isopropanol alcohol (IPA) and dried by a continuous flow of nitrogen gas. Sb_2_Te_3_ etching was then carried out by inductively‐coupled‐plasma (ICP, Oxford Instruments Plasmalab System 100), with a RF power of 100 watts, ICP power of 500 watts, HBr with a flow rate of 50 sccm (standard‐cubic‐centimeters‐per‐minute), Ar with a flow rate of 5 sccm under a process pressure of 5 mTorr, and a temperature of 50 °C.

### Photolithography, Dry Etching, and Metal Contact Fabrication

The metasurface sample was coated with hexamethyldisilizane (HMDS) to enhance the adhesion of the photoresist with the sample surface. Then the sample was spin‐coated with photoresist, S1811 at 3000 rpm. After spin coating, the sample undergoes a soft‐baking process for 1 min at 100 °C. This results in a photoresist layer with a thickness of ≈1.5 µm. After that, the sample was aligned under the desired pattern drawn on a chromium mask, using the mask aligner system (EVG6200). Ultraviolet light with a dose of 50 mJ cm^−^^2^ was exposed to the photoresist. Finally, the sample was developed with MF319 for 1.5 min to create the pattern. Sb_2_Te_3_ strip was etched by using inductively‐coupled‐plasma (ICP, Oxford Instruments Plasmalab System 100) with the same condition in dry Etching of Sb_2_Te_3_ metasurface. Then the remaining photoresist was cleaned by using acetone. The procedure of photolithography was the same as the mask creation of Sb_2_Te_3_ strip. Here, the chromium mask for electrode contact was used. 20‐nm‐thick Ti was deposited by electron beam evaporation (Denton Explorer), at a deposition rate of 1 angstrom per second, and at a pressure of 9×10^−7^ Torr. Then, 60 nm Au was deposited using the same system, at a deposition rate of 1 angstrom per second, and at a pressure of 9×10^−7^ Torr.

### Characterizations

The experimental mid‐IR spectra were obtained using a microscope‐based Fourier‐transform infrared (FTIR, Bruker Hyperion 2000) spectroscopy system, operating in reflection mode. The system employed a globar as the illumination source, providing a stable and broad‐spectrum infrared light necessary for accurate spectral analysis. The detection of the reflected infrared light was carried out using a mercury cadmium telluride (MCT) detector, which was cooled with liquid nitrogen to enhance its sensitivity and to reduce its thermal noise, thereby improving the accuracy and reliability of the measurements. To investigate the polarization properties of the samples, a wire‐grid polarizer was incorporated into the experimental setup. The visible reflectance spectra of Sb_2_Te_3_ nanodisk arrays were measured using a Craic micro‐spectrometer, with a ×5 objective lens with a numerical aperture of 0.12. Moreover, the optical images of the color palettes were captured by an Olympus microscope (MX61) using the “analySIS” software. The objective was a ×10 MPlanFLN, NA = 0.30 lens. Before the image was captured, a white color balancing was carried out on a 100‐nm‐thick aluminum film, which was prepared by electron beam evaporation. SEM images were taken at an acceleration voltage of 10 keV with the Elionix, ESM‐9000 SEM.

The visible photoresponse was measured by using WITec Alpha 300 S Optical Microscope in confocal mode. A 532 nm CW laser was focused on the sample. The generated photovoltage was then recorded by sourcemeter (Keithley 2636B). The mid‐IR photoresponse was measured by using a homemade photocurrent measurement system where the infrared light with different polarization status was obtained from a serious quantum cascade lasers (Daylight Solutions, MIRcat) and tunable wavelength in the range of 4–8 µm combining a serious half‐wave plate and quarter‐wave plate, and then focused on the samples using a zinc selenide IR focusing lens with a focal length of 50 mm. The generated photovoltage was then recorded by a highly sensitive source meter unit (Keysight, B2912A).

### Numerical Simulations

The optical simulations and power absorption density of the Sb_2_Te_3_ nanodisk arrays were performed using a Lumerical finite‐difference time‐domain (FDTD) solver. A plane source with wavelengths between 380 nm and 11 µm was input in the negative *z*‐direction perpendicular to the nanodisk array and substrate. A field monitor was placed 100 nm above the plane source to measure the reflected power, while another field monitor was placed perpendicular to the substrate along *x* = 0 nm to measure the electric and magnetic fields in the cross‐section of the nanodisk. Periodic boundary conditions were set for the *x*‐ and *y*‐boundaries, while the perfectly matched layer (PML) boundary condition was set for the *z*‐boundaries. Refractive index data for the polycrystalline phase of Sb_2_Te_3_ were measured using an ellipsometer as shown in Figure 1b. Power absorption density was calculated by *P*_abs_  =  1/2*ωε*″″ | *E* |^2^, and was normalized by *P*_0_, the incident power divided by the meta‐molecule volume. FDTD simulations were used to calculate the absorption power density of Sb_2_Te_3_ nanostructures, which were then input into COMSOL to simulate the resulting heat conduction and temperature distribution. Multipole decomposition analysis and thermal simulation were carried out using the COMSOL Multiphysics 5.6 Optical Wave Optics Module and heat transfer modules, which exploits the Finite Element Method. Because of differences in length scales, first electromagnetic field simulations were computed with smaller simulation regions, and absorbed power was input into a heat transfer simulation that had a much larger simulation area. A single unit‐cell of the structure was simulated using periodic boundary conditions under normally incident plane‐wave.

## Conflict of Interest

The authors declare no conflict of interest.

## Author Contributions

J.K.W.Y. and Z.D. conceived the concepts and supervised the project. R.E.S. conceived the concept of mid‐IR Sb_2_Te_3_ detector. S.Z., S.A., J.Z., H.L.Y.L. and Z.D. prepared the samples. S.Z. and J.Z. did the SEM characterizations and interpretations. S.A. and S.Z. did the electron beam lithography, dry etching and developed the whole fabrication processes. M.D. and Q.J.W. did the mid‐IR device characterizations and interpretations. S.Z. and Y.L. did numerical simulations. The authors thank Dr. Wei Chen from Xiamen University for the support on the simulations. N.Q.A. did the sputtering of Sb_2_Te_3_ films, under the guidance of R.E.S. Y.L. and S.Z. did thermal simulation and analysis. S.Z. and Q.Y.S.W. did the optical reflectance measurements. S.A., Y.L. and Z.D. provided expertise in data analysis and interpretations. N.L.M.W. did mid‐IR ellipsometer measurement and interpretation. A.S. did the characterization of the Seebeck coefficient and interpretation. J.D. participated in the discussions and provided the suggestions. The paper was drafted by S.Z with input from Z.D., J.K.W.Y. and S.A. All authors analyzed the data and read and corrected the manuscript before the submission. S.Z. and S.A. are equal contributions.

## Supporting information



Supporting Information

## Data Availability

The data that support the findings of this study are available from the corresponding author upon reasonable request.
