# Supplementary material for: Chalcogenide Metasurfaces Enabling Ultra‐Wideband Detectors From Visible to Mid‐infrared
Source: Adv Sci (Weinh). 2025 Feb 19;12(14):2413858. doi: 10.1002/advs.202413858 (PMC11984864; doi:10.1002/advs.202413858)
Supplement: Supplementary file 1 — Supporting Information [file ADVS-12-2413858-s001.pdf]

ADVANCED SCIENCE
Open Access

Supporting Information

for *Adv. Sci.*, DOI 10.1002/advs.202413858

Chalcogenide Metasurfaces Enabling Ultra-Wideband Detectors From Visible to Mid-infrared

Shutao Zhang, Shu An, Mingjin Dai, Qing Yang Steve Wu, Nur Qalishah Adanan, Jun Zhang, Yan Liu, Henry Yit Loong Lee, Nancy Lai Mun Wong, Ady Suwardi, Jun Ding, Robert Edward Simpson, Qi Jie Wang*, Joel K. W. Yang* and Zhaogang Dong**

Supplementary Information for

Chalcogenide Metasurfaces Enabling Ultra-Wideband Detectors from Visible to Mid-infrared

Shutao Zhang[1,2,3,#], Shu An[1,#], Mingjin Dai[4], Qing Yang Steve Wu[1], Nur Qalishah Adanan[2], Jun Zhang[1], Yan Liu[1], Henry Yit Loong Lee[1], Nancy Lai Mun Wong[1], Ady Suwardi[1,5], Jun Ding[3], Robert Edward Simpson[2,6,*], Qi Jie Wang[4,*], Joel K. W. Yang[2,*] and Zhaogang Dong[1,2,3,*]

[1]Institute of Materials Research and Engineering (IMRE), Agency for Science, Technology and Research (A*STAR), 2 Fusionopolis Way, Innovis #08-03, Singapore 138634, Republic of Singapore

[2]Singapore University of Technology and Design (SUTD), 8 Somapah Road, 487372, Singapore

[3]Department of Materials Science and Engineering, National University of Singapore, 9 Engineering Drive 1, Singapore 117575

[4]School of Electrical and Electronic Engineering, Nanyang Technological University, Singapore 639798, Singapore

[5]Department of Electronic Engineering, The Chinese University of Hong Kong, Sha Tin, New Territories, Hong Kong SAR 999077, China

[6]University of Birmingham, Edgbaston, B15 2TT, UK

[#]These authors equally contribute to this work.

*Correspondence and requests for materials should be addressed to J.K.W.Y. (email: joel_yang@sutd.edu.sg), Q.J.W. (email: qjwang@ntu.edu.sg), R.E.S. (email: r.e.simpson.1@bham.ac.uk) and Z.D. (email: zhaogang_dong@sutd.edu.sg).

Table S1: Comparison of key performance metrics of thermoelectric detectors in recent studies.

Figure S1: Schematic illustration of the designed Sb_2Te_3 nanodisk.

Figure S2: Characterization of Sb_2Te_3 film.

Figure S3: The refractive index (n) and extinction coefficient (k) of Sb_2Te_3 in visible region.

Figure S4: Simulated absorptance spectrum of Sb_2Te_3 films with varying thickness.

Figure S5: Multi-physical decomposition of the Sb_2Te_3 metasurface interactions.

Figure S6: The refractive index (n) and extinction coefficient (k) of Sb_2Te_3 in near-IR range.

Figure S7: Simulated temperature gradient for the thermoelectric detector without the Sb_2Te_3 metasurface.

Figure S8: Simulated potential gradient for the thermoelectric detector without the Sb_2Te_3 metasurface.

Figure S8: Simulated relationship between temperature difference, incident power, and photovoltage in Sb_2Te_3 detector.

Figure S9: Simulation results on the relationship between temperature difference, incident power, and photovoltage across the two electrodes of the Sb_2Te_3 detector.

Figure S10: Nanofabrication process for Sb_2Te_3 thermoelectric detectors.

Figure S11: Photograph of the actual Sb_2Te_3 detector device.

Figure S12: Measured reflectance of Sb_2Te_3 mid-IR metasurface with various diameters.

Figure S13: Characterization of photodetector response time based on 10-90 % rise and fall times of Sb_2Te_3 detector at 0.1 mW.

Figure S14: Optimization workflow for Sb_2Te_3 nanostructures using particle swarm optimization (PSO).

Figure S15: Simulated absorptance spectrum of linear polarization-selective Sb_2Te_3 devices under varying polarization angles.

Figure S16: The absorption intensity distribution of simulated Sb_2Te_3 polarization selective metasurface cross-section ($|P/P_0|$).

Figure S17: Schematic diagram of experimental device for photovoltage measurement.

Table S1. Comparison of key performance metrics of thermoelectric detectors in recent studies.

Active materials	Enhancement mechanism	Spectral range	Responsivity	Ref.
**Sb_2Te_3**	**Metasurface enhance**	**0.532-10 μm**	**24 V/W**	**This work**
Graphene	Seebeck coefficient manipulation	0.6 μm	1.5 mA/W	[1]
$PdSe_2$	Asymmetric contact	4.6-10 μm	13 V/W	[2]
$PdSe_2$	Asymmetric contact	8.6 μm	-	[3]
$PdSe_2$	Hot-electrons assisted	4.6-10.5 μm	13 V/W	[2]
BP	Doping enhance	3.4 THz	3 V/W	[4]
Bi_2Te_2Se/ Sb_2Te_3	Doping enhance	0.514 μm	-	[5]
SnSe	Doping enhance	0.44-1.55 μm	33 mV/W	[6]
Perovskite/ Graphene	Composite materials enhance	0.98 μm	44.24 mV/W	[7]
PbTe/CdTe	Composite materials enhance	1.5-4.0 μm	0.1 A/W	[8]

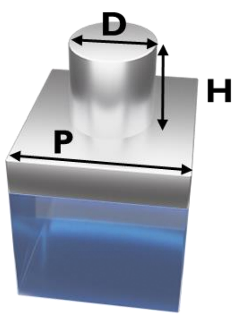

Fig. S1. Schematic illustration of the designed Sb_2Te_3 nanodisk. The designed array contains pitch(P), height(H) and diameter(D), with 150 nm Sb_2Te_3 base layer.

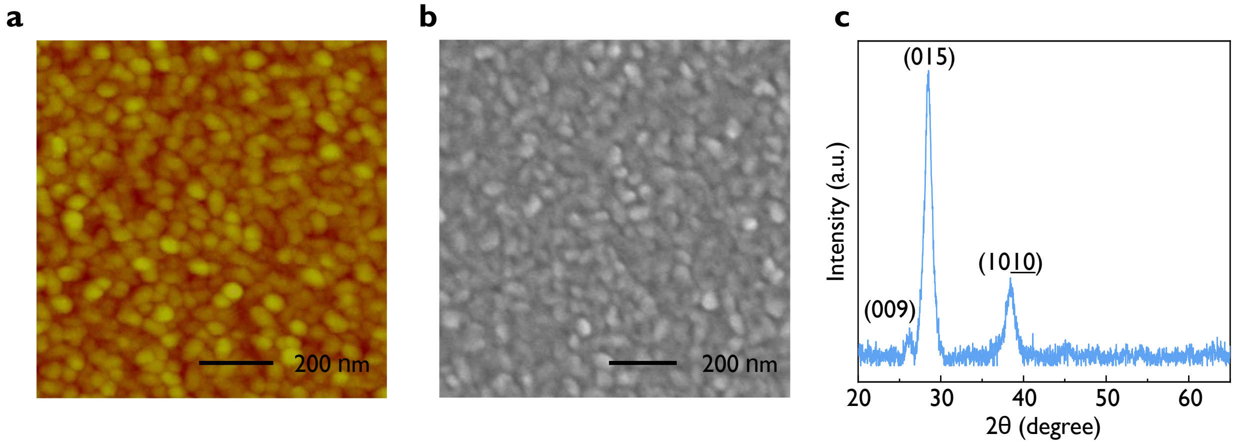

Fig. S2. Characterization of Sb_2Te_3 film. (a) AFM images of the Sb_2Te_3 as-deposited film, showing a surface roughness of 3.2 nm. (b) SEM images of the as-deposited film. (c) XRD characterization of the as-deposited film, indicating that the layered structure of Sb_2Te_3 exhibits a pronounced (015) orientation.

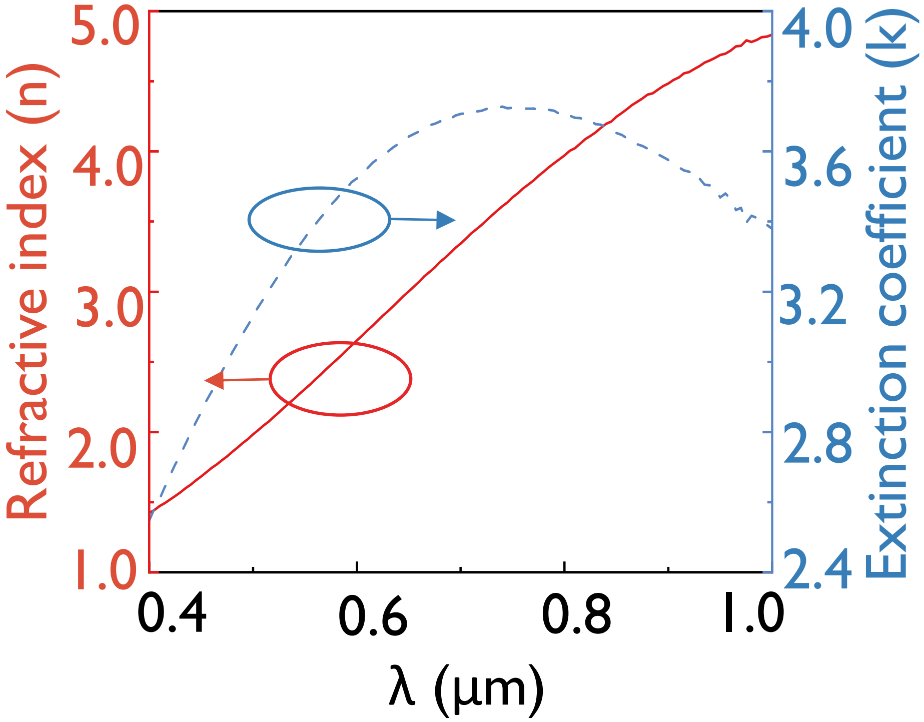

Fig. S3. The refractive index (n) and extinction coefficient (k) of Sb_2Te_3 in visible region.

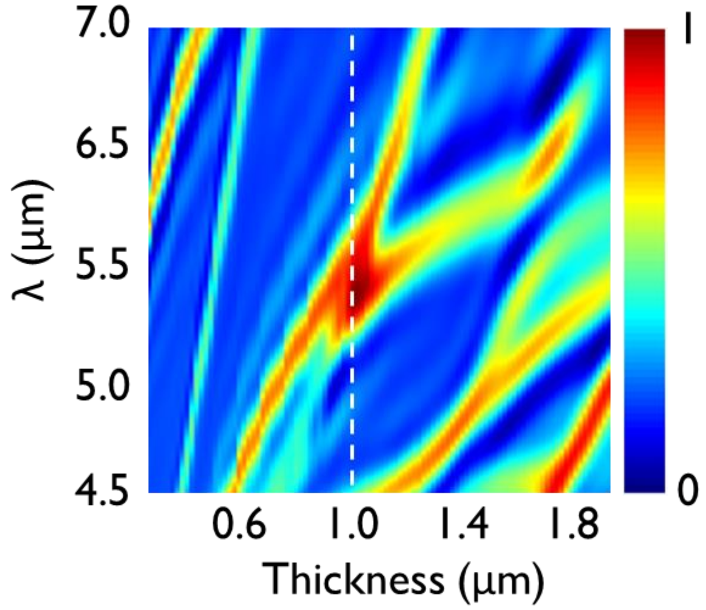

Figure S4. Simulated absorptance spectrum of Sb_2Te_3 films with varying thickness.

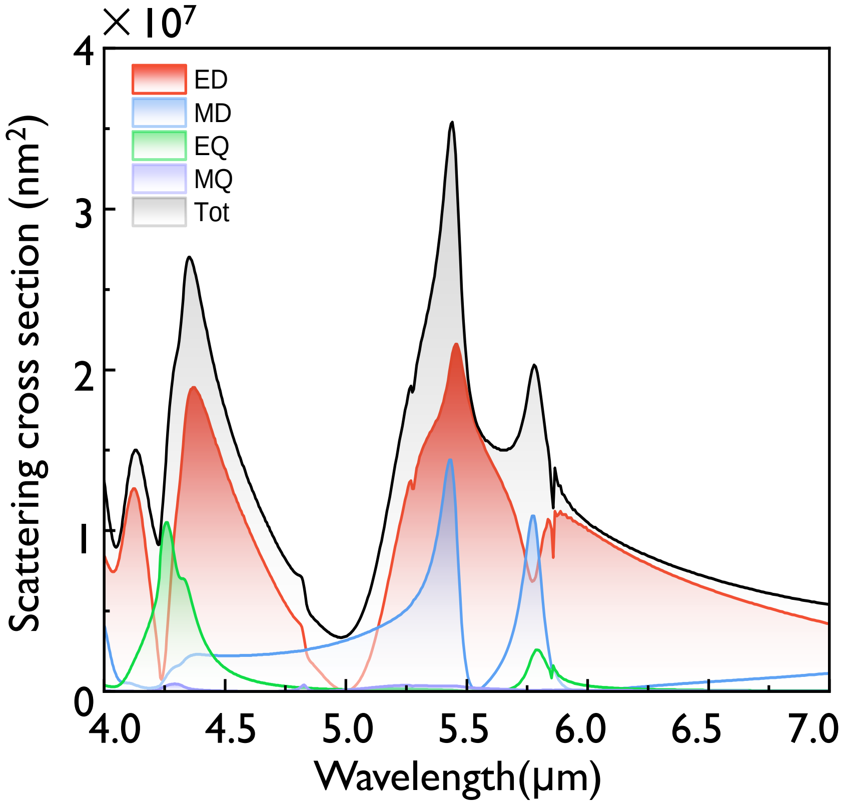

Fig. S5. Multi-physical decomposition of the Sb_2Te_3 metasurface interactions. Showing contributions of electric dipole (ED), magnetic dipole (MD), electric quadrupole (EQ), magnetic quadrupole (MQ), and the total combined effect (Tot).

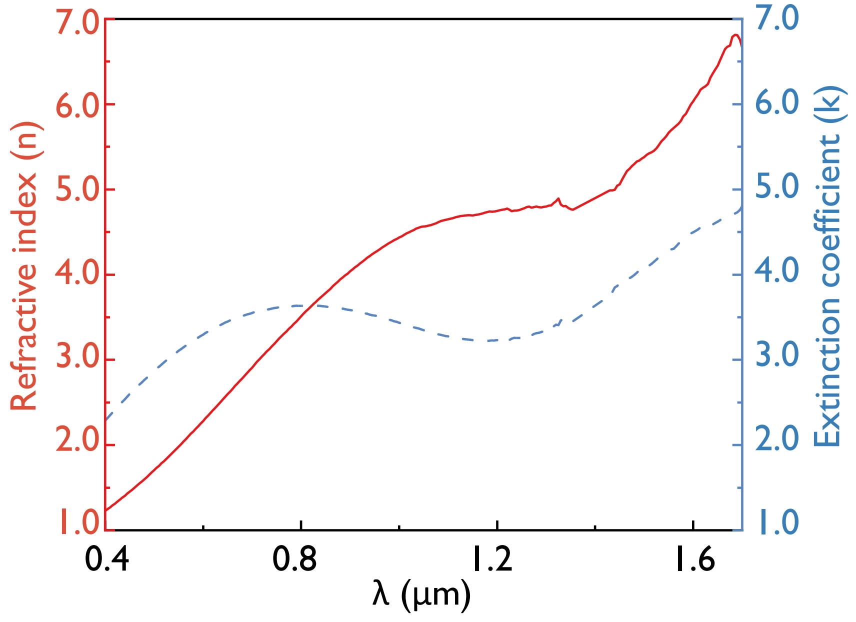

Fig. S6. The refractive index (*n*) and extinction coefficient (*k*) of Sb_2Te_3 in near-IR range.

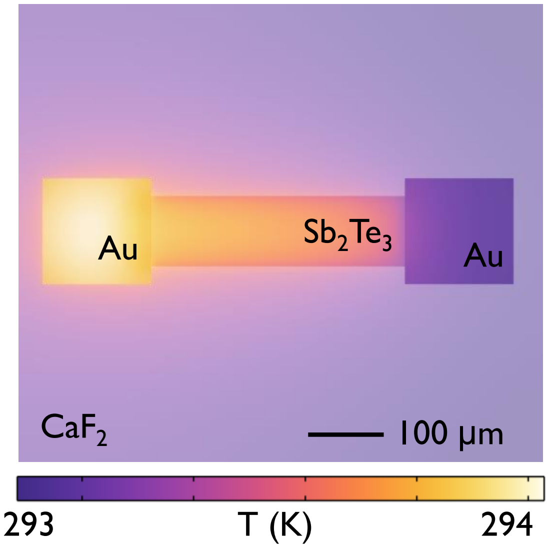

Fig. S7. Simulated temperature gradient for the thermoelectric detector without the Sb_2Te_3 metasurface. Simulated temperature gradient with an input power of 0.1 mW.

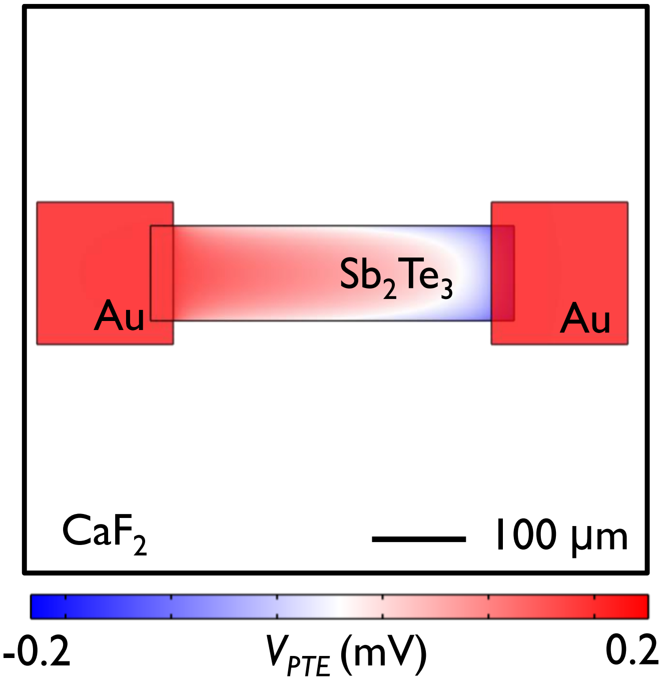

Fig. S8. Simulated potential gradient for the thermoelectric detector without the Sb_2Te_3 metasurface. Simulated potential with an input power of 0.1 mW.

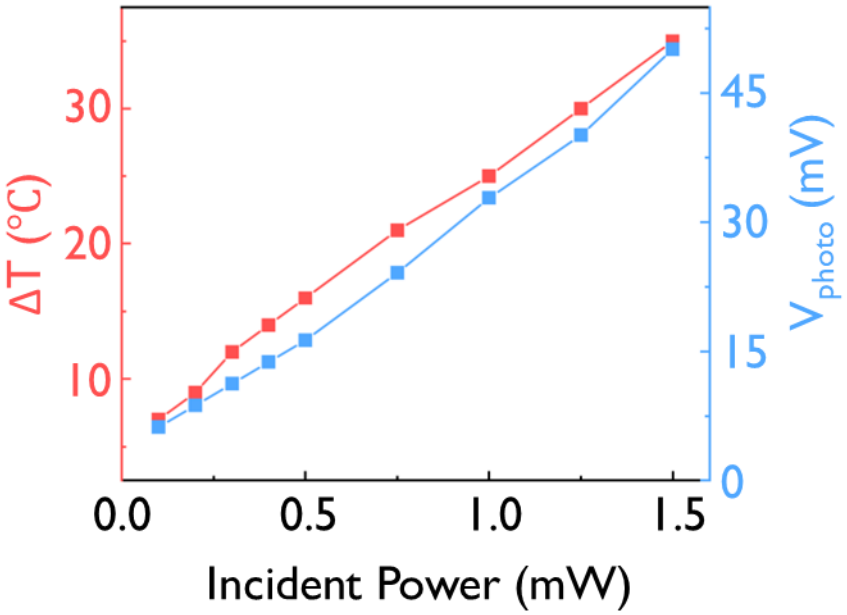

Fig. S9. Simulation results on the relationship between temperature difference, incident power, and photovoltage across the two electrodes of the Sb_2Te_3 detector.

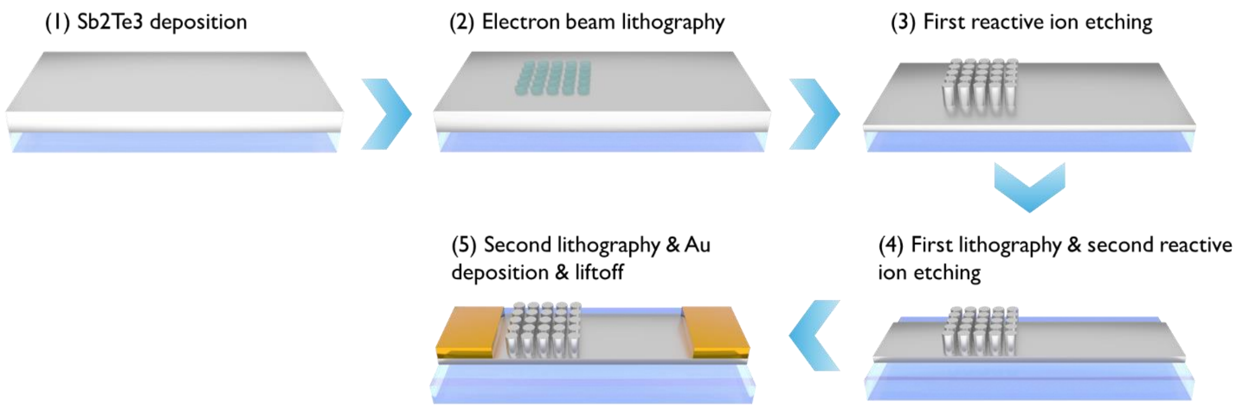

Fig. S10. Nanofabrication process for Sb_2Te_3 thermoelectric detectors. (1) Deposition of Sb_2Te_3 thin film using RF sputtering. (2) E-beam exposure to fabricate the HSQ resist mask. (3) Inductively-coupled plasma (ICP) for silicon etching with HBr/Ar gases. (4) lithography exposure and ICP etching to fabricate the Sb_2Te_3 strip. (5) Deposition of Au electrodes using thermal or electron-beam evaporation. Integration of the device with PCB board and connections for testing.

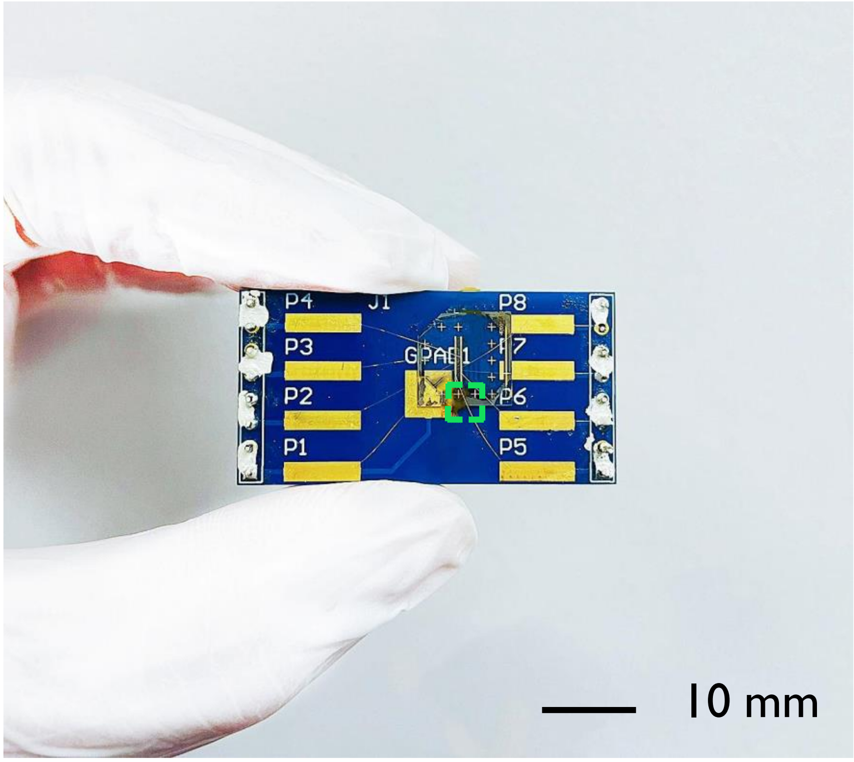

Fig. S11. Photograph of the actual Sb_2Te_3 detector device. Wirebonded to pads on a printed circuit board (PCB), and an optical microscope image of the Sb_2Te_3 photodetector in the center panel.

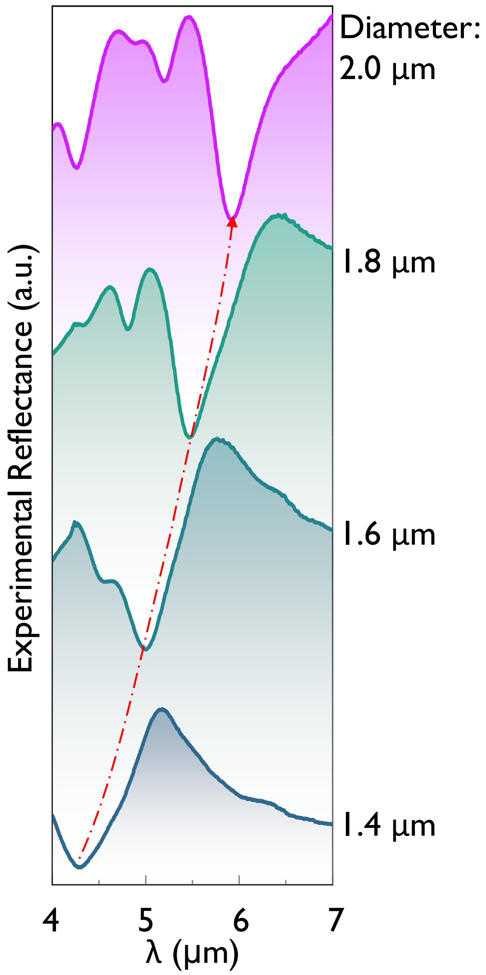

Fig. S12. Measured reflectance of nanodisks with various diameters. (Diameter = 1.4-2.0 μm, step size = 0.2 μm).

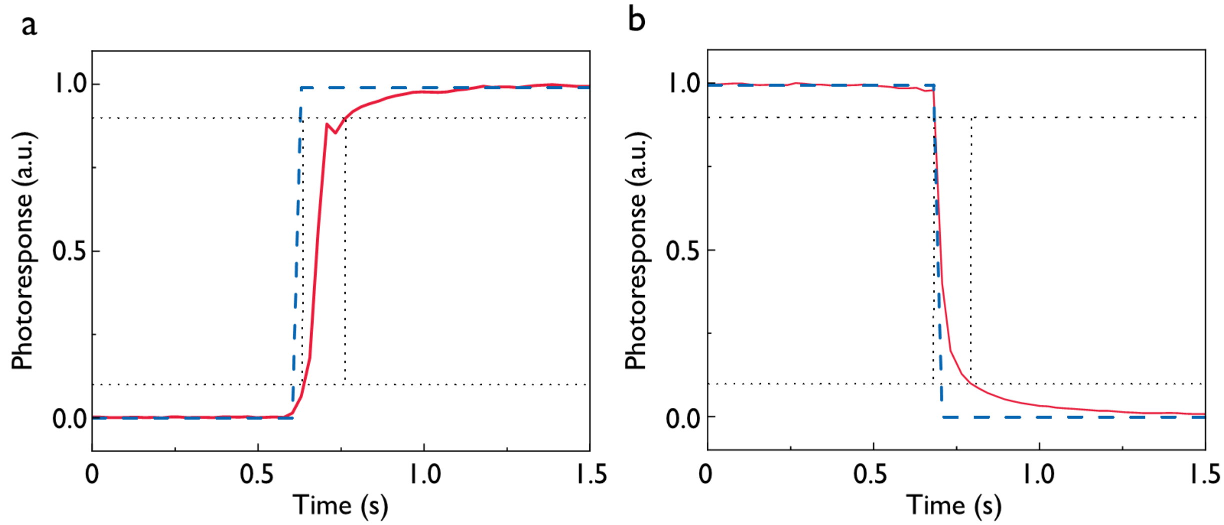

Fig. S13. Characterization of photodetector response time based on 10-90 % rise and fall times of Sb_2Te_3 detector at 0.1 mW. Red solid lines are representative of the Sb_2Te_3 detector response.

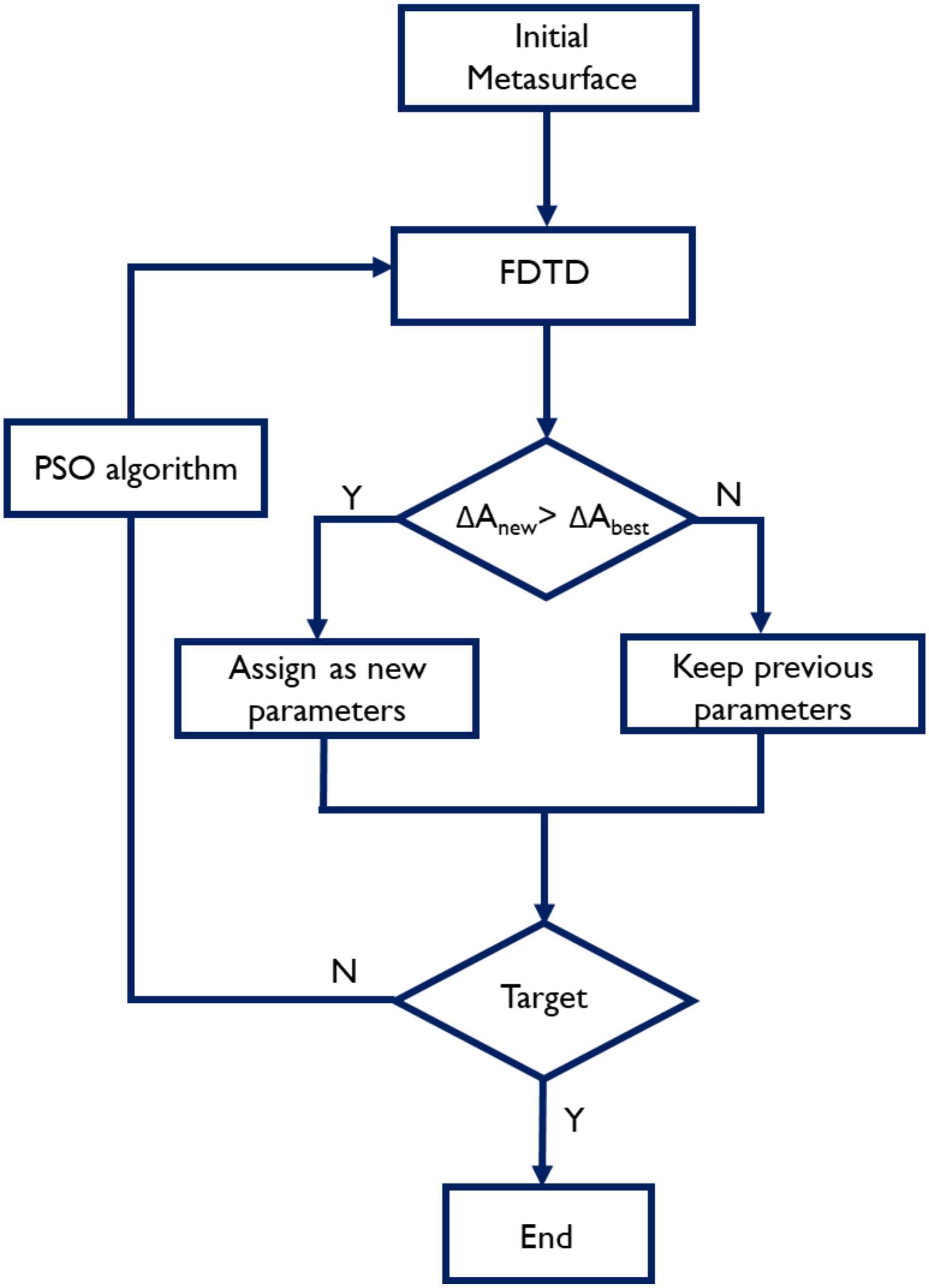

Fig. S14. Optimization workflow for Sb_2Te_3 nanostructures using particle swarm optimization (PSO).

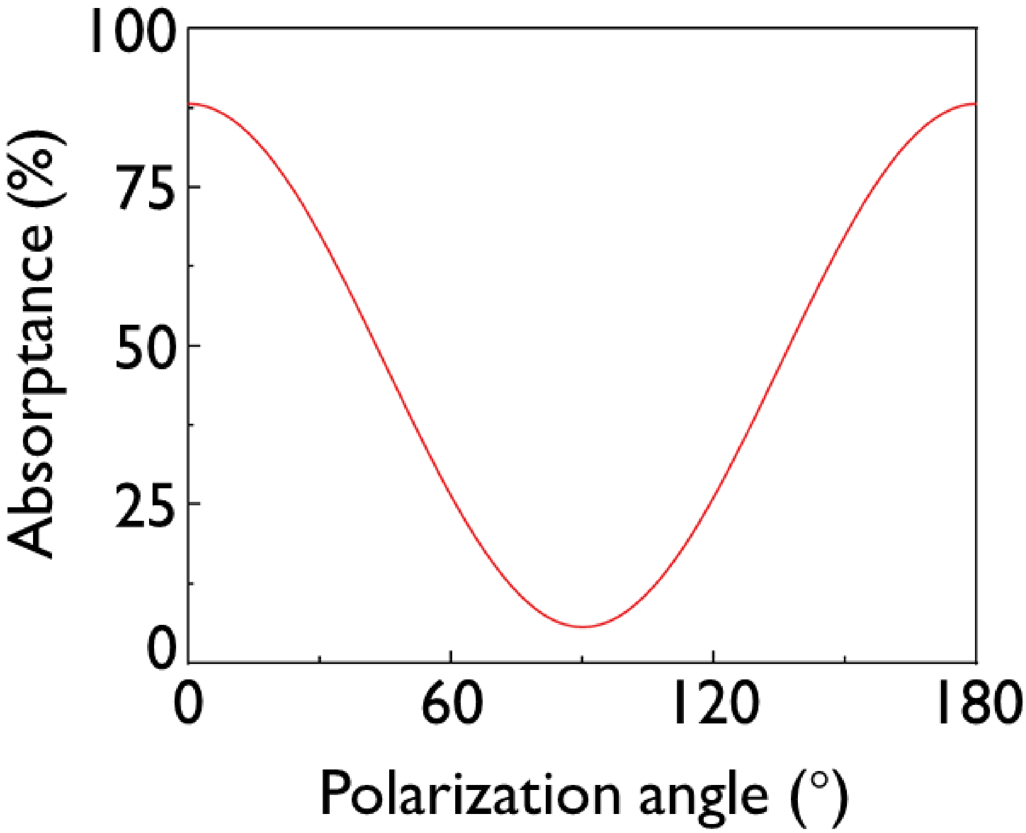

Fig. S15. Simulated absorptance spectrum of linear polarization-selective Sb_2Te_3 devices under varying polarization angles.

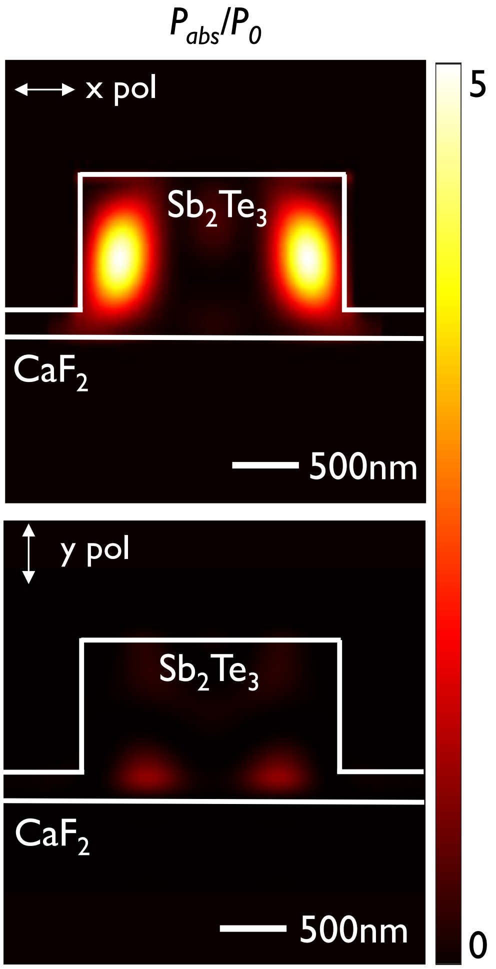

Fig. S16. The absorption intensity distribution of simulated Sb_2Te_3 polarization selective metasurface cross-section (|*P/P_0*|) at 4.5 µm.

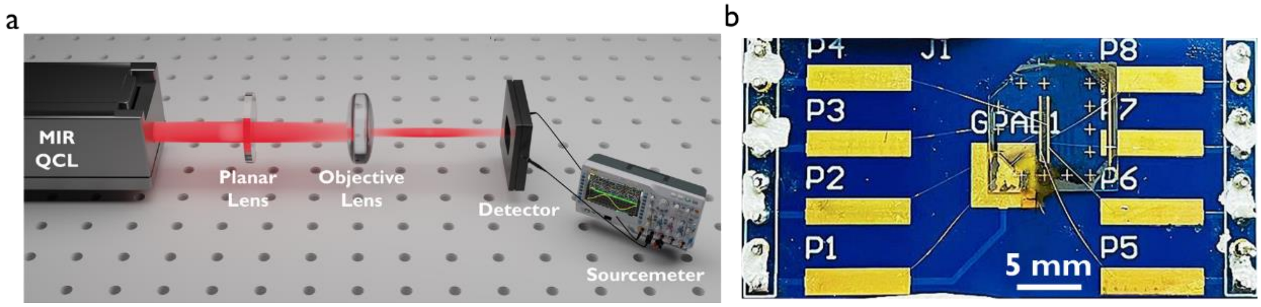

Fig. S17. (a) Schematic illustration on the experimental device for photovoltage measurement. (b) Optical microscope image of the Sb_2Te_3 detector with the wire bonded electrodes.

Reference:

[1] M. C. Lemme, F. H. Koppens, A. L. Falk, M. S. Rudner, H. Park, L. S. Levitov, C. M. Marcus, *Nano letters* **2011**, 11, 4134.

[2] M. Dai, C. Wang, M. Ye, S. Zhu, S. Han, F. Sun, W. Chen, Y. Jin, Y. Chua, Q. J. Wang, *ACS nano* **2022**, 16, 295.

[3] V. A. Semkin, A. V. Shabanov, D. A. Mylnikov, M. A. Kashchenko, I. K. Domaratskiy, S. S. Zhukov, D. A. Svintsov, *Nano Letters* **2023**, 23, 5250.

[4] L. Viti, A. Politano, K. Zhang, M. S. Vitiello, *Nanoscale* **2019**, 11, 1995.

[5] S. Mashhadi, D. L. Duong, M. Burghard, K. Kern, *Nano Letters* **2017**, 17, 214.

[6] L.-D. Chen, H.-T. Jiang, C.-H. Yin, E.-R. Zhang, Y.-Y. Hou, X.-L. Zhou, F. Wang, Y.-Y. Lv, X.-J. Yan, J. Zhou, *Applied Physics Letters* **2023**, 123.

[7] M. Li, X. Tang, S. Wang, T. Li, J. Li, H. Zhao, Q. Li, Q. Wang, Y. Zhang, J. Yao, *Ceramics International* **2022**, 48, 4366.

[8] S. Chusnutdinow, S. Schreyeck, S. Kret, A. Kazakov, G. Karczewski, *Applied Physics Letters* **2020**, 117.
